# Zerumbone-incorporated liquid crystalline nanoparticles inhibit proliferation and migration of non-small-cell lung cancer in vitro

**DOI:** 10.1007/s00210-023-02603-5

**Published:** 2023-07-13

**Authors:** Bikash Manandhar, Keshav Raj Paudel, Dvya Delilaa Clarence, Gabriele De Rubis, Thiagarajan Madheswaran, Jithendra Panneerselvam, Flavia C. Zacconi, Kylie A. Williams, Lisa G. Pont, Majid Ebrahimi Warkiani, Ronan MacLoughlin, Brian Gregory Oliver, Gaurav Gupta, Sachin Kumar Singh, Dinesh Kumar Chellappan, Philip M. Hansbro, Kamal Dua

**Affiliations:** 1 Discipline of Pharmacy, Graduate School of Health, University of Technology Sydney, Sydney, NSW 2007 Australia; 2 Australian Research Centre in Complementary and Integrative Medicine, Faculty of Health, University of Technology Sydney, Sydney, NSW 2007 Australia; 3 Centre for Inflammation, Centenary Institute and University of Technology Sydney, Faculty of Science, School of Life Sciences, Sydney, NSW 2050 Australia; 4 School of Postgraduate Studies, International Medical University (IMU), 57000 Kuala Lumpur, Malaysia; 5 Department of Pharmaceutical Technology, School of Pharmacy, International Medical University, 57000 Kuala Lumpur, Malaysia; 6 Departamento de Química Orgánica, Facultad de Química y de Farmacia, Pontificia Universidad Católica de Chile, Av. Vicuña Mackenna 4860, 7820436 Macul, Santiago Chile; 7 Centro de Investigación en Nanotecnología y Materiales Avanzados, CIEN-UC, Pontificia Universidad Católica de Chile, Av. Vicuña Mackenna 4860, 7820436 Macul, Santiago Chile; 8 Institute for Biological and Medical Engineering, Schools of Engineering, Medicine and Biological Sciences, Pontificia Universidad Católica de Chile, Santiago, Chile; 9 School of Biomedical Engineering, University of Technology Sydney, Sydney, New South Wales Australia; 10 Institute for Biomedical Materials and Devices, Faculty of Science, University of Technology Sydney, Sydney, New South Wales Australia; 11 Research and Development, Aerogen Limited, IDA Business Park, Galway, Connacht, H91 HE94 Ireland; 12 School of Pharmacy & Biomolecular Sciences, Royal College of Surgeons in Ireland, Dublin, Leinster, D02 YN77 Ireland; 13 School of Pharmacy & Pharmaceutical Sciences, Trinity College, Dublin, Leinster, D02 PN40 Ireland; 14 Woolcock Institute of Medical Research, Macquarie University, Sydney, NSW 2137 Australia; 15 School of Life Sciences, Faculty of Science, University of Technology Sydney, Sydney, NSW 2007 Australia; 16 School of Pharmacy, Suresh Gyan Vihar University, Jaipur, Rajasthan India; 17 Center for Transdisciplinary Research, Saveetha Institute of Medical and Technical Science, Saveetha University, Chennai, India; 18 School of Pharmaceutical Sciences, Lovely Professional University, Jalandhar-Delhi G.T Road, Phagwara, 144411 India; 19 Department of Life Sciences, School of Pharmacy, International Medical University, 57000 Kuala Lumpur, Malaysia

**Keywords:** Zerumbone, Liquid crystalline nanoparticles, Non-small-cell lung cancer, A549 lung cancer cells, Cell proliferation, Cell migration

## Abstract

Lung cancer is the second most prevalent type of cancer and is responsible for the highest number of cancer-related deaths worldwide. Non-small-cell lung cancer (NSCLC) makes up the majority of lung cancer cases. Zerumbone (ZER) is natural compound commonly found in the roots of *Zingiber zerumbet* which has recently demonstrated anti-cancer activity in both in vitro and in vivo studies. Despite their medical benefits, ZER has low aqueous solubility, poor GI absorption and oral bioavailability that hinders its effectiveness. Liquid crystalline nanoparticles (LCNs) are novel drug delivery carrier that have tuneable characteristics to enhance and ease the delivery of bioactive compounds. This study aimed to formulate ZER-loaded LCNs and investigate their effectiveness against NSCLC in vitro using A549 lung cancer cells. ZER-LCNs, prepared in the study, inhibited the proliferation and migration of A549 cells. These inhibitory effects were superior to the effects of ZER alone at a concentration 10 times lower than that of free ZER, demonstrating a potent anti-cancer activity of ZER-LCNs. The underlying mechanisms of the anti-cancer effects by ZER-LCNs were associated with the transcriptional regulation of tumor suppressor genes *P53* and *PTEN*, and metastasis-associated gene *KRT18*. The protein array data showed downregulation of several proliferation associated proteins such as AXL, HER1, PGRN, and BIRC5 and metastasis-associated proteins such as DKK1, CAPG, CTSS, CTSB, CTSD, and PLAU. This study provides evidence of potential for increasing the potency and effectiveness of ZER with LCN formulation and developing ZER-LCNs as a treatment strategy for mitigation and treatment of NSCLC.

## Introduction

Lung cancer represents one of the deadliest respiratory diseases that accounted for 1.7 million deaths worldwide in 2020 (Sung et al. 2021). Non-small-cell lung cancer (NSCLC) comprises of the majority of the cases (~85% of lung cancer cases) (Malyla et al. 2020). Conventional treatment strategies for NSCLC face limitations of disease relapse occurrences, resistance to chemotherapeutic treatment (Baci et al. 2022; Li et al. 2022), and elevated toxicity resulting from chemotherapy and radiotherapy (Kumbhar et al. 2022; Yazbeck et al. 2022).

There is an emerging interest in nanotechnological applications for delivering anti-cancer drugs to achieve a higher drug specificity and therapeutic efficacy. Liquid crystalline nanoparticles (LCNs) have unique structural properties that can add benefits to anti-cancer therapy by providing high capacity of loading lipophilic and hydrophilic drugs, low toxicity, controlled release, nontoxic degradation, biodegradable matrix, low cost and ease of scale up for topical, oral, and intravenous drug administration (Jain et al. 2012; Pardeike et al. 2009). The LCNs also provide the added benefits of site-specific drug delivery system, modulated drug release, and long-term stability (Muller et al. 2002). Nanotechnology and LCNs have been successfully employed to incorporate and deliver several plant-derived phytoceuticals, including berberine (Alnuqaydan et al. 2022; Mehta et al. 2021; Paudel et al. 2022), curcumin (Clarence et al. 2022; Sharma et al. 2021), rutin (Paudel et al. 2021), naringenin (Wadhwa et al. 2021), boswellic acid (Solanki et al. 2020), and agarwood oil (Alamil et al. 2022) to improve their therapeutic potency and efficacy.

Zerumbone (ZER) is a bioactive compound isolated from the *Zinigiber zerumbet* (Girisa et al. 2019) that has previously shown to exert potent anti-inflammatory (Su et al. 2021), anti-oxidant (Sidahmed et al. 2015), and anti-cancer activities (Foong et al. 2018; Ghasemzadeh et al. 2017; Rahman et al. 2014). ZER can induce apoptosis in A549 lung cancer cells and enhance cisplatin treatment efficacy in vitro (Hu et al. 2014). ZER can also inhibit proliferation of lung cancer cells in vivo mouse models (Kim et al. 2009). However, its medicinal applications are hindered by its poor aqueous solubility and bioavailability. With incorporation into LCN drug delivery system, ZER has potential for its development as an alternative anti-cancer therapy that can effectively inhibit lung cancer proliferation and migration as well as resolve the issues of drug toxicity, adverse effects, and treatment resistance that are apparent with the conventional therapy. In the present study, ZER was formulated into a monoolein-based LCNs, and the resulting ZER-loaded LCNs (ZER-LCNs) were characterized for physiochemical properties including mean size, entrapment efficiency and in vitro release of ZER. The anti-cancer potential of ZER-LCNs was then tested in vitro in human A549 adenocarcinoma cell line in comparison with that of free ZER.

The results of this study provide substantial evidence of the advantages and feasibility of encapsulating poorly soluble bioactive molecule ZER in LCNs-based nano drug delivery systems for improving their anti-cancer potency and efficacy against NSCLC.

## Materials and methods

### Materials

ZER (MW218.3) was purchased from Funakoshi Co., Ltd (originally produced by Adipogen Life Science, Japan), Monoolein (MO, 1-oleoyl-rac-glycerol, MW 356.55 g/mol, purity 99.5%), Poloxamer 407 (P407), and phosphate buffered saline (PBS) containing 137 mM sodium chloride, 2.7 mM potassium chloride, and 10 mM phosphate buffer were purchased from Merck (Kenilworth, NJ, USA). Ultrapure water and reverse-osmosis (RO) purified water were used in several studies. All the solvents and reagents used in the study were of analytical research grade.

### Preparation and characterization of ZER-LCNs

#### Solubility analysis of ZER

For the solubility analysis, 10 mg of ZER was added with 1mL of methanol to obtain a concentration of 10000 μg/mL. This was then further diluted with methanol to obtain concentrations of 10, 20, 30, 40, 50, 60, 70, and 80 μg/mL. The concentration of soluble ZER in these samples was determined by measuring their absorbance at a wavelength (λ) of 249 nm using the UV-Vis. The optical density (OD) values of the samples were then plotted against their concentration and the R2 value (greater than 0.99) as well as the graph equation was obtained.

#### Preparation of ZER-LCN formulation

The ZER-LCN formulation was prepared by the ultrasonification method, with the formulation composition, as specified in Table 1. The method was conducted by briefly heating MO at 60 °C in a water bath. ZER was then added into the glass vial containing the molten MO, then gently shaken until it was completely dissolved. Simultaneously, P407 was added to water and heated at 60 °C in a water bath. After a brief period, the P407 that was dissolved in the water was added to the ZER-MO mixture. After this, the coarse dispersion obtained was subjected to size reduction by sonication for 5 min and using the ultrasonic cell pulverizer (Labsonic® P; Sartorius) at amplitude 80 and repeated cycles of 5 s on and 5 s off.

**Table 1:** Formulation composition of blank LCNs and ZER-LCNs

Formulation	Formulation composition (%w/w)			
code	MO	P407	Distilled water	ZER
Blank-LCN	4.00	0.40	95.60	0.00
ZER-LCN	4.00	0.40	95.58	0.02

#### Entrapment efficiency

0.1 mL of ZER-LCN was added to 0.9 mL of methanol to obtain 10 mL of sample. The absorbance of total ZER (free ZER+ZER entrapped in LCNs) in the sample was measured using the UV-Vis method at λ=249 nm. The concentration of total ZER in the sample was calculated by comparing the absorbance against the standard curve obtained from the solubility analysis. In order to obtain the concentration of free ZER (non-entrapped ZER) in the ZER-LCN formulation, 2 mL of the sample was transferred to the Amicon Ultra-4 centrifugal filter device (molecular-weight-cut off: 10,000 g/mol; Merck Millipore Ltd., Cork, Ireland), and centrifuged for 15 min at 2800xg, 25 °C to separate the free drug from ZER-LCNs. The supernatant was collected, and the absorbance was measured using the UV-Vis method at λ=249 nm. The concentration of free ZER in the supernatant was calculated by comparing the absorbance against the standard curve obtained from solubility analysis. The entrapment efficiency (%EE) was calculated using the following equation.

$$\%\mathrm{EE}\;=\;(\mathrm{Total}\;\mathrm{ZER}\;\mathrm{concentration}\;-\mathrm{Free}\;\mathrm{ZER}\;\mathrm{concentration})/\mathrm{Total}\;\mathrm{ZER}\;\mathrm{concentration}\;\times\;100\%$$

#### In vitro release study

**Fig. 1:**
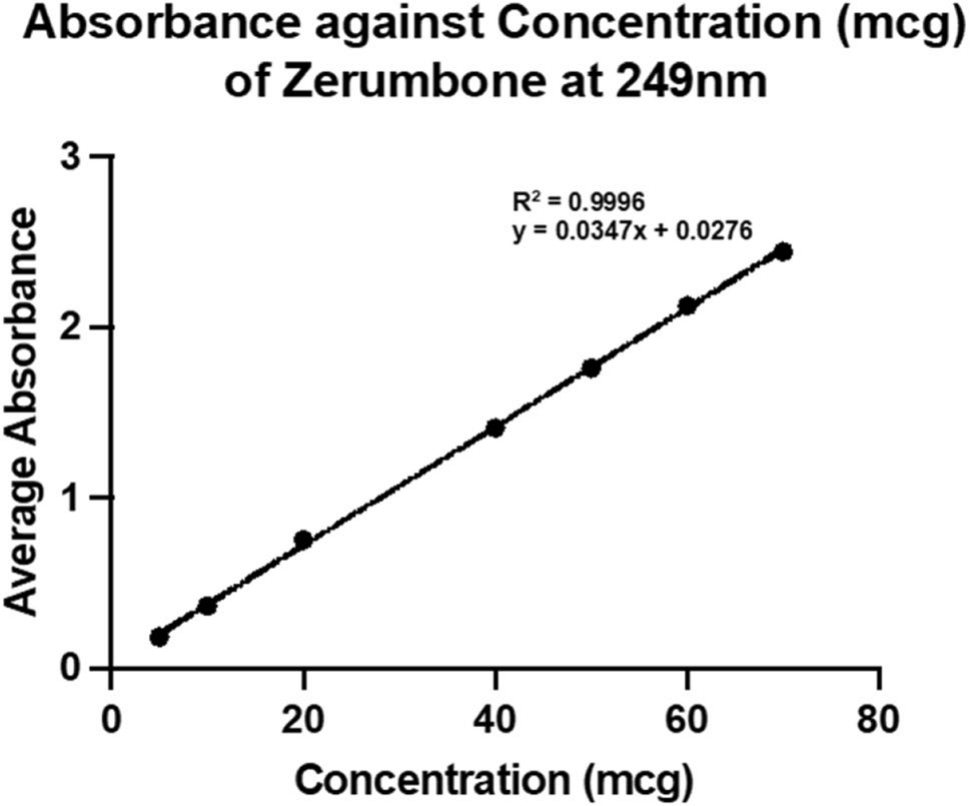
Graph of absorbance against concentration of Zerumbone

The release profiles of ZER from ZER-LCN formulations were observed using the static dialysis method dialysis tubing cellulose membrane; molecular-weight-cut-off: 14,000 g/mol; Merck). By using the quantification of absorbance, the amount of drug released was tested while being immersed in PBS (pH7.4). The dialysis bags were soaked in water prior to use and were then filled with 1 mL of ZER-LCN sample before being clamped at both ends and submerged in 20 mL of PBS (pH 7.4) in a 50 mL centrifuge tube. The samples in the centrifuge tubes were then submerged into a water-bath at 37 °C (SW22 Julabo, shaken horizontally at 50 strokes per min), thus mimicking the intestinal milieu. Then, 1 mL of each sample was drawn from the tubes at intervals 1, 2, 3, 6, 9, 12, and 24 h. Every time 1 mL of sample was drawn, the same volume was replenished with PBS to ensure that the volume is remained constant. The concentration of ZER in the drawn samples was quantified by measuring absorbance using UV-spectrometry and comparing against the standard curve (Fig. 1).

The cumulative released percentage of ZER was calculated using the equation:

$$\mathrm{Cumulative}\;\mathrm{drug}\;\mathrm{release}\;(\%)\;=\;(\mathrm{CiV}+\mathrm{Ve}\;(\mathrm C9\mathrm i-1)+\mathrm C(\mathrm i-2)+\cdots.+\mathrm C1)/\mathrm T\;\times\;100\%$$

Where, Ci represents the ‘i'th sampling concentration of ZER (μg/mL), V is the total the volume of release medium (mL), Ve is the sampling volume (mL), and T is the total mass of ZER present in nanoparticles (μg).

### Cell culture and treatment

Human lung carcinoma A549 cells (ATCC, Manassas, VA, USA) was kindly gifted by Prof. Alaina Ammit (Woolcock Institute of Medical Research, Sydney, Australia). The cells were cultured in low-glucose Dulbecco’s Modified Eagle’s Medium (DMEM, Lonza, Basel, Switzerland), 5% (v/v) fetal bovine serum (Lonza) and 1% (v/v) antibiotic mix of penicillin and streptomycin (Lonza) in a humidified incubator. The incubator was maintained with 5% CO_2_ at 37 ºC. The in vitro experiments were carried out by treating A549 cells with ZER or ZER-LCNs at the indicated doses for 24 h.

### Cell proliferation assay

A549 cell proliferation was measured using 3-(4,5-dimethylthiazol-2-yl)-2,5-diphenyl tetrazolium bromide (MTT, Merck), as previously described (Manandhar et al. 2020). Briefly, the cells were incubated for 24 h in the absence or presence of ZER-LCNs (1, 2.5, 5, 7.5, or 10 µM) or ZER (1, 5, 10, 25, or 50 µM). MTT (250 µg/mL) was added, and the plate was incubated for 4 h. The supernatant was discarded and 100 µL dimethyl sulfoxide (Merck) was added to dissolve the purple formazan crystals. The absorbance was measured at 540 nm excitation wavelength with the help of TECAN Infinite M1000 plate reader (Tecan Trading AG, Männedorf, Switzerland). The cell proliferation was measured as % cell viability relative to control.

### Colony formation assay

Colony formation assay was performed as previously described (Alnuqaydan et al. 2022). Briefly, A549 cells were plated at a density of 500 cells per well in 6-well plates and cultured at 37 °C for two weeks in the absence or presence of 50 µM ZER or 5 µM ZER-LCNs, with repeated replenishment of the media every 48 h. The cells were washed with PBS and fixed at room temperature for 20 minutes with 3.7% (v/v) formaldehyde. After three washes with PBS, the cells were stained with 0.4% crystal violet (Merck). The cells were washed again three times with PBS. The individual wells were photographed from the bottom side of the plates.

### Wound healing assay

The impact of ZER and ZER-LCNs on A549 cell migration was evaluated using a wound healing experiment. A549 cells were seeded at a density of 2.5×10^5^/well in 6-well plates and cultured until fully confluent. The cell monolayer was scratched with the tip of a sterile pipette to create a wound. After washing the cells with PBS to remove floating cells, the cells were cultured at 37 °C for 24 h with or without ZER (50 µM) or ZER-LCNs (2.5 or 5 µM). At 0 and 24 h of incubation, images were captured using a phase contrast microscope equipped with a 10× objective lens. The wound closure was determined as a percentage (%) of the change in wound width between 0 h and 24 h relative to that at 0 h.

### Trans-well chamber migration assay

The A549 cell migration was also evaluated using a trans-well chamber migration assay as described previously (Paudel et al. 2022).

### Reverse transcriptase-quantitative polymerase chain reaction (RT-qPCR)

After 24 h of incubation of A549 cells in the presence or absence of ZER (50 µM) or ZER-LCNs (2.5 or 5 µM), the cells were washed twice with PBS and lysed with TRI reagent (Merck). The total RNA isolation was conducted as previously described (Alnuqaydan et al. 2022). The RNA purity and concentration was determined using Nanodrop One (Thermo Fisher Scientific). The RNA was reverse transcribed to synthesize cDNA and qPCR was performed to determine the mRNA levels of genes of interest (Alnuqaydan et al. 2022). The forward and reverse primers for genes encoding tumor protein 53 (*P53*, forward: ACCTATGGAAACTACTTCCTG; reverse: ACCATTGTTCAATATCGTCC), phosphatase and tensin homolog (*PTEN*, forward: GGCTAAGTGAAGATGACAATC; reverse: GTTACTCCCTTTTTGTCTCTG), keratin 18 (*KRT18*, forward: GGAAGTAAAAGGCCTACAAG; reverse: GTACTTGTCTAGCTCCTCTC), and glyceraldehyde-3-phosphate dehydrogenase (*GAPDH*, forward: TCGGAGTCAACGGATTTG; reverse: CAACAATATCCACTTTACCAGAG) were procured from Merck.

### Proteome profiler human oncology array

After 24 h of incubation of A549 cells with or without ZER (50 µM) or ZER-LCNs (2.5 or 5 µM), the proteins were extracted with RIPA buffer ((Roche Diagnostics, Basel, Switzerland). The proteins were quantified by bicinchoninic acid (BCA) assay using Pierce BCA protein assay kit (Thermo Fisher). Three hundred micrograms protein per group was used to assess the expression of oncology-related proteins using proteome profiler human XL oncology array kit (R&D Systems, Minneapolis, MN), according to the manufacturer’s instructions. The protein signals in the array were photographed using the ChemDoc MP imaging system (Bio-Rad, Hercules, CA, USA). The Image J software (version 1.53c, Bethesda, MD, USA) was used to analyze the pixel densities of the protein signals.

### Statistical analysis

The data are presented as mean ± S.E.M and statistical analyses were performed by one-way ANOVA, followed by Dunnett’s multiple comparison tests using GraphPad Prism software (version 9.4, San Diego, CA, USA). A *p*-value of less than 0.05 was considered statistically significant.

## Results

### Characterization of ZER-LCN formulation

Based on the solubility analysis, it was found that the λmax for ZER was at 249 nm. After which a concentration against absorbance study was conducted and a graph was developed from it which indicated that the absorbance increased in a linear concentration dependent manner as observed in Fig. 1. The linear equation of y = 0.0347x + 0.0276 and an R^2^ value of 0.9996 was obtained from the graph which indicates that there is a strong and significant correlation.

**Fig. 2:**
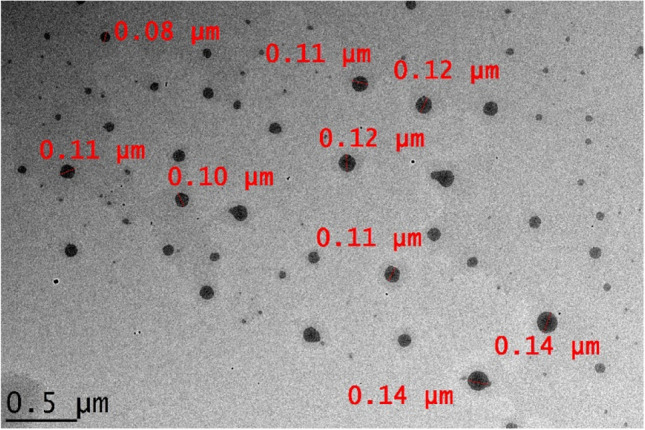
The analysis of the surface morphologies as visualized on the transmission electron microscopy (Hitachi HT7700 high resolution TEM; Hitachi, Chiyoda, Tokyo, Japan). Scale bar 0.5 µm

The prepared ZER-LCNs was a white-pigmented viscous liquid. As seen in Fig. 2, the TEM analysis which functioned to show the surface morphology of the formulation showed that the LCNs without ZER had clear cuboidal particles and the similar morphological results were observed when ZER was added into the LCNs. Both samples showed less than 200 nm in size, which indicated positive results to the mean particles size measurement as the smaller the particle size, the better the targeting ability of the drug delivery system.

**Fig. 3:**
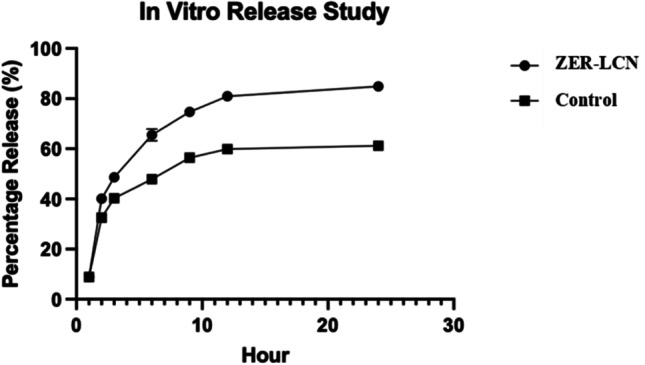
The in vitro release study of free ZER (control) and ZER-LCNs

The mean diameter of ZER-LCN was determined to be 180.6 ± 3 nm with all formulations showing a narrow PDI of less than 0.4 while also being negatively charged. Adding on to that, high entrapment efficiency was also observed for the ZER-LCN formulation at 90.63 ± 0.13% together with a prolonged release over a duration of 24 h. Besides that, the results for the in vitro release test shown in Fig. 3 suggested that the ZER-LCN formulation had a greater amount of drug release in comparison to the free ZER. It was also observed and confirmed that ZER-LCN was also able to produce a drug release over a prolonged period, which in this study was up to 24 h. However, it was observed that there was an initial quick and rapid release which was eventually followed by a plateau at about slightly more than 80% in the ZER-LCN in comparison to the free ZER which plateaued at around 60%.

### Effects of ZER and ZER-LCNs on cell proliferation of A549 cells

**Fig. 4:**
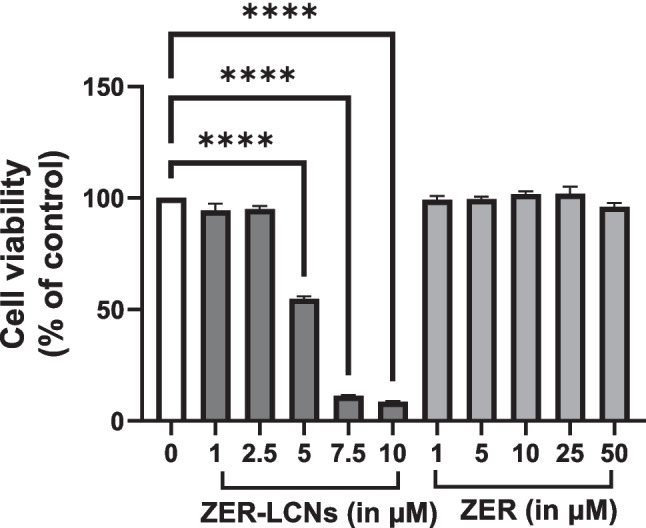
Anti-proliferative effects of ZER and ZER-LCNs in A549 cells. A549 cells were treated with or without ZER-LCNs (1, 2.5, 5, 7.5, or 10 µM) or ZER (1, 5, 10, 25, or 50 µM) for 24 h, followed by incubation with MTT. The purple formazan crystals formed were dissolved with DMSO and the absorbance was measured with microplate reader. The data in the figure are mean ± SEM of 3 independent experiments. ****p<0.0001

ZER did not show inhibitory effect on the cell proliferation of A549 cells up to a concentration of 50 µM. ZER-LCNs, on the other hand, exerted ~45% inhibition of A549 cell proliferation at a dose of 5 µM (Fig. 4, *p*<0.0001 compared to the control). The inhibition of cell proliferation by ZER-LCNs increased up to ~90% at doses of 7.5 and 10 µM (Fig. 4, *p*<0.0001 for both compared to the control). ZER at 50 µM and ZER-LCNs at 2.5 or 5 µM were used for subsequent experiments to evaluate their anti-cancer potential on A549 cells.

### ZER-LCNs reduced colony formation of A549 cells

**Fig. 5:**
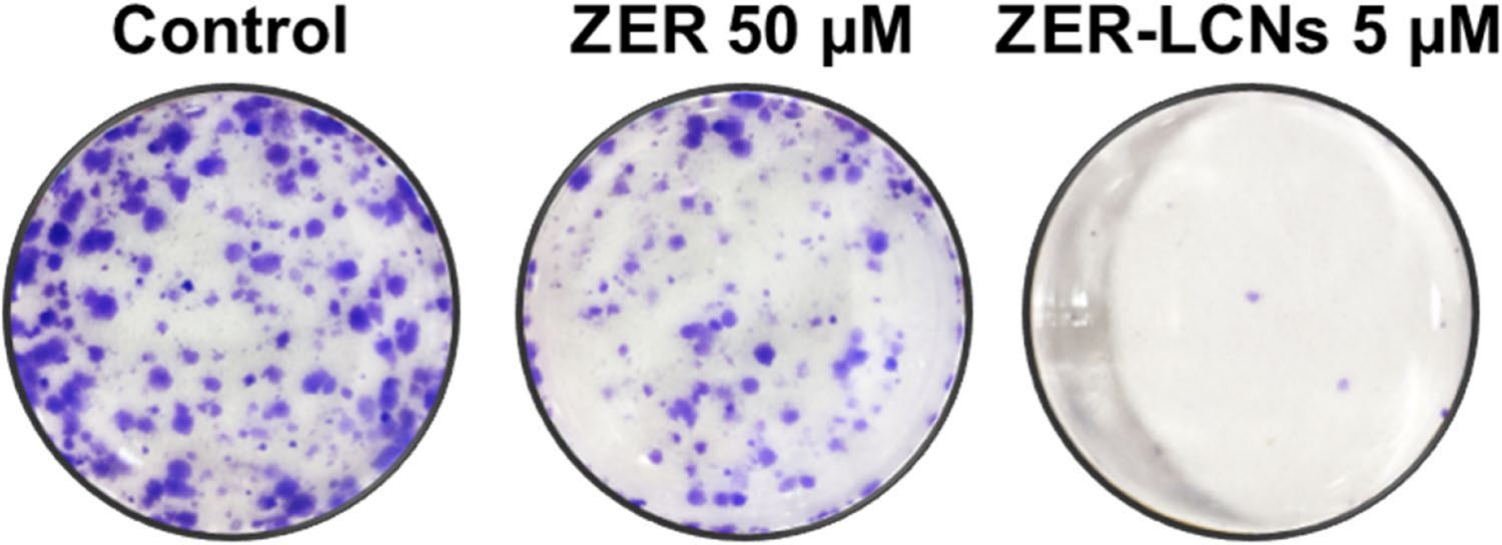
Effect of ZER and ZER-LCNs on colony formation of A549 cells. After seeding A549 cells in 6-well plate, they were cultured for 2 weeks in the absence or presence of 50 µM ZER or 5 µM ZER-LCNs. The cell colonies were stained with crystal violet and photographed. The figure shows representative images from 3 independent experiments

The crystal violet staining of the A549 cell colonies clearly demonstrates the anti-proliferative activities of both ZER (50 µM) and ZER-LCNs (5 µM), as compared to the control (Fig. 2). The representative well images visibly depict a modest decrease in the number of cell colonies in ZER-treated cells while almost no colonies in ZER-LCNs-treated cells (Fig. 5), suggesting a greater potency of nanoparticle formulation of ZER compared to free ZER.

### ZER-LCNs inhibited wound closure in A549 cells

**Fig. 6:**
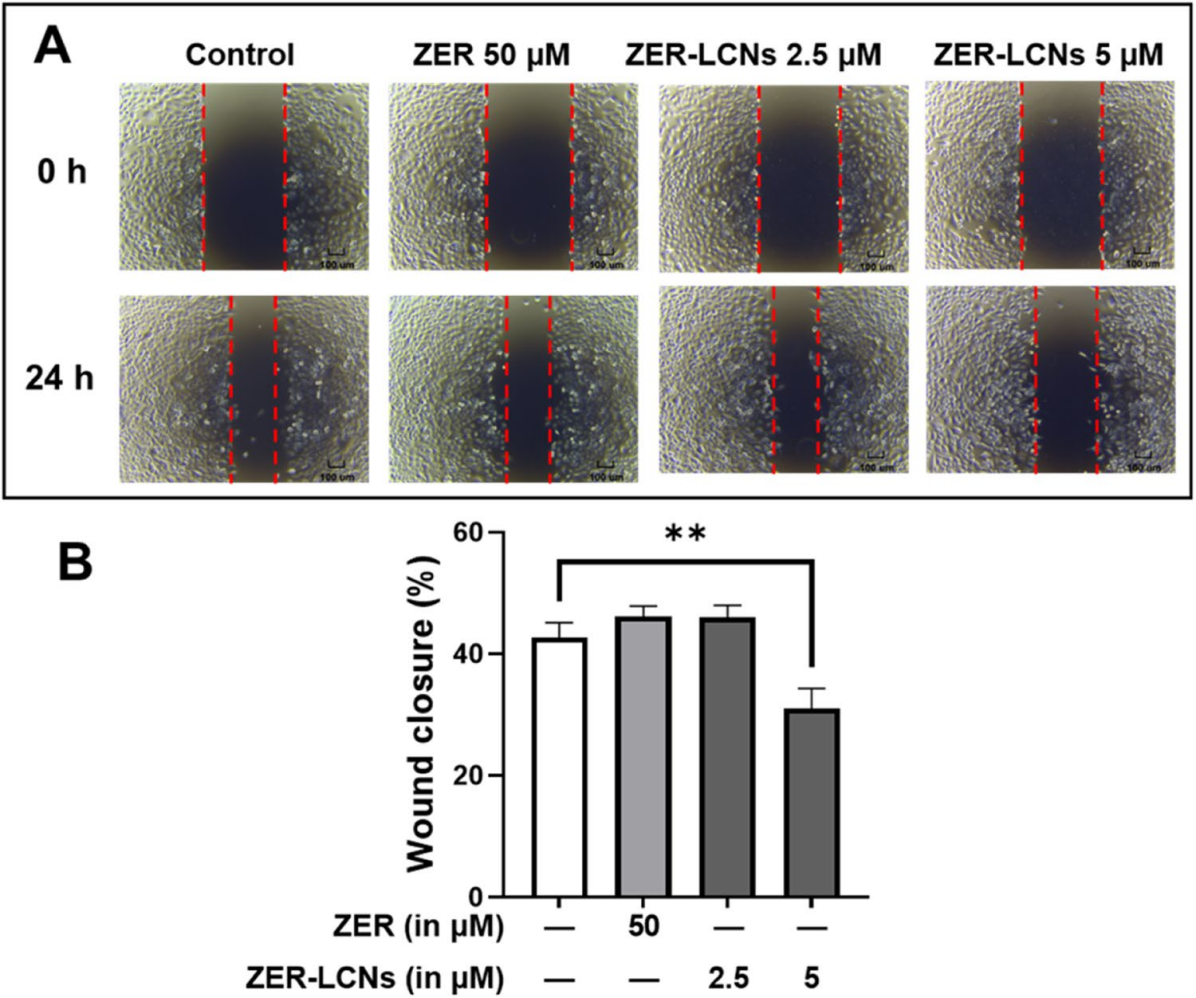
Wound healing effect of ZER and ZER-LCNs in A549 cells. Wound was scratched on the monolayer of fully confluent A549 cells, then the cells were cultured for 24 h in the absence or presence of 50 µM ZER, 2.5 µM ZER-LCNs, or 5 µM ZER-LCNs. The images were acquired at 0 h and 24 h of treatment with a phase contrast microscope at 4× magnification. **A** representative images from 3 independent experiments. **B** % wound closure after 24 h of incubation with or without ZER-LCNs. Data in **B** is expressed as mean ± SEM of 3 independent experiments. **p<0.01

The images from wound healing assay show no difference in the closure of wound in A549 cells treated with ZER (50 µM) or ZER-LCNs (2.5 µM) (Fig. 6A). However, there is a visible inhibition of wound closure in A549 cells treated with ZER-LCNs at 5 µM concentration (Fig. 6A). The quantification of % wound closure is consistent with the representative images, with ~27% inhibition of wound closure in 5 µM ZER-LCNs-treated A549 cells as compared to the control (Fig. 6B, p<0.01).

### ZER-LCNs decreased the migration of A549 cells in a trans-well chamber

**Fig. 7:**
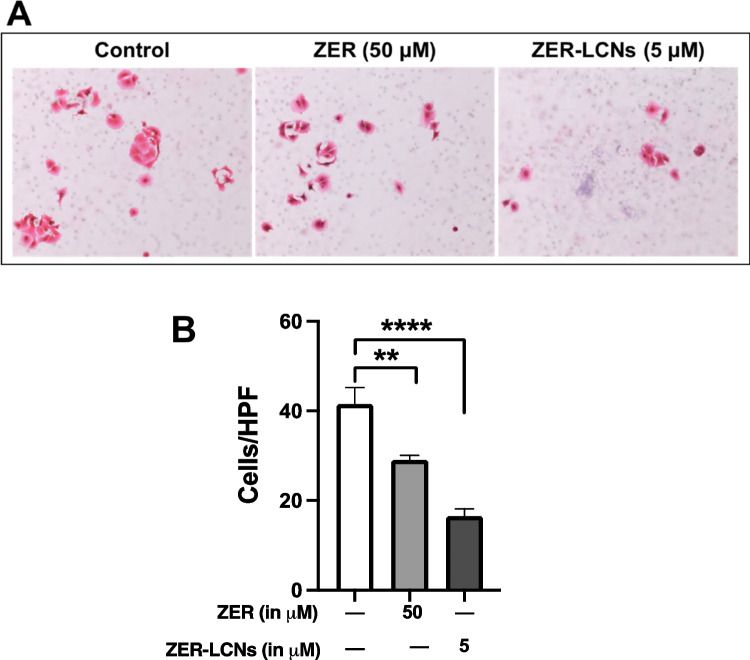
Effect of ZER and ZER-LCNs on migration of A549 cells in a trans-well chamber. The cells seeded in a trans-well chamber was treated with/without 50 µM ZER or 5 µM ZER-LCNs for 24 h to allow cell migration. Migrated cells in the lower compartment were stained with staining solution of hematoxylin and eosin and microscopic images were taken at 20× magnification. **A** representative images from 3 independent experiments. **B** The number of cells, and the data are expressed as mean ± SEM of 3 independent experiments. **p<0.01, ****p<0.0001

The visual observation of images from trans-well chamber assay showed decreased number of A549 cell migration when treated with 50 µM ZER or 5 µM ZER-LCNs compared to the control (Fig. 7A). This is also consistent with the decreased number of migrated cells in both 50 µM ZER-treated group (Fig. 7B, p<0.01 against the control) and 5 µM ZER-LCNs (Fig. 7B, p<0.0001 against the control). Fifty micromolar ZER and 5 µM ZER-LCNs decreased cell migration by ~32% and ~60%, respectively (Fig. 7B), suggesting a greater anti-migratory potential of ZER-LCNs compared to the free ZER.

### ZER-LCNs increased P53 and PTEN mRNA expression and decreased KRT18 mRNA expression

**Fig. 8:**
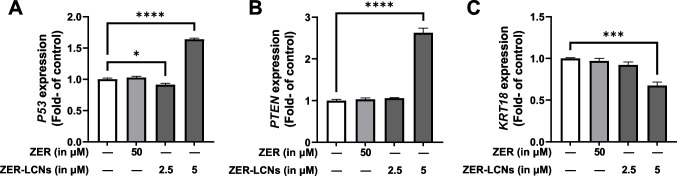
Regulation of mRNA levels *P53*, *PTEN*, and *KRT18* by ZER and ZER-LCNs. A549 cells were treated with 50 µM ZER, 2.5 µM ZER-LCNs, or 5 µM ZER-LCNs for 24 h. Total RNA was extracted, cDNA synthesized, and mRNA levels were determined with qPCR. The figure shows the mRNA levels of *P53* (**A**), *PTEN* (**B**) and *KRT18* (**C**), normalized against the levels of *GAPDH*. Data are expressed as mean ±SEM of 3 independent experiments. *p<0.05, ***p<0.001, ****p<0.0001

Analysis of mRNA levels with RT-qPCR showed that 5 µM ZER-LCNs upregulates the transcription of tumor suppressor genes *P53* and *PTEN* (Fig. 8A–B, p<0.0001 for both against the control) and downregulates transcription of metastasis-associated *KRT18* (Fig. 8C, p<0.001). Fifty micromolar ZER and 2.5 µM ZER-LCNs did not show such effects (Fig. 8A–C).

### ZER-LCNs downregulated proteins-associated with cancer cell proliferation

**Fig. 9:**
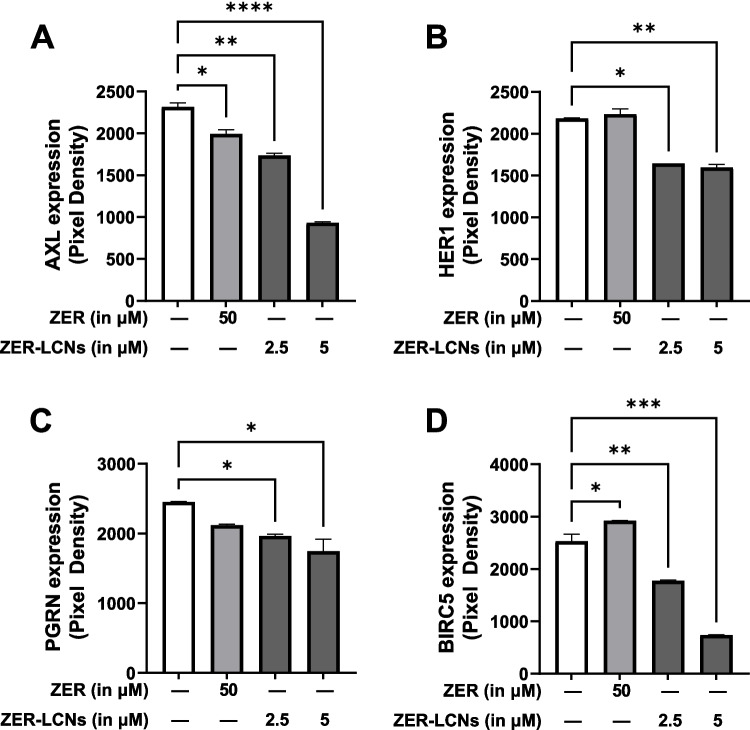
Regulation of expression of proteins-related to cancer cell proliferation by ZER and ZER-LCNs. After treatment of A549 cells with 50 µM ZER, 2.5 µM ZER-LCNs, or 5 µM ZER-LCNs for 24 h, the proteins were extracted and the expression of proteins-associated with cell proliferation was evaluated with proteome profiler human oncogenic array kit. The figure shows the protein expression of AXL (**A**), HER1 (**B**), PGRN (**C**), and BIRC5 (**D**). The data are expressed as mean ± SEM of 3 independent experiments. *p<0.05, **p<0.01, ***p<0.001, and ****p<0.0001

Treatment of A549 cells with ZER-LCNs significantly downregulated the protein expression of AXL receptor tyrosine kinase (AXL), epidermal growth factor receptor (HER1), progranulin (PGRN), and baculoviral IAP repeat containing 5 (BIRC5) at both 2.5 and 5 µM concentrations (Fig. 9A–D), while ZER only downregulated the protein expression of AXL (Fig. 9A, p<0.05 compared to control). This shows that the regulatory activity of ZER-LCNs on expression of proteins associated with cancer cell proliferation was far superior compared to ZER (Fig. 9A–D).

### ZER-LCNs downregulated proteins-associated with cancer cell migration

**Fig. 10:**
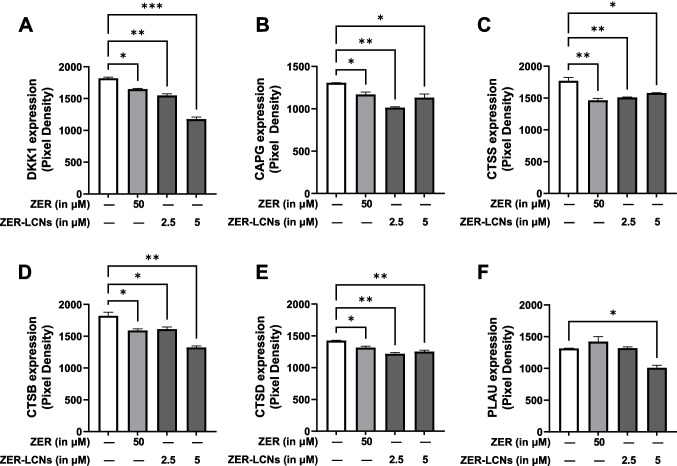
Regulation of proteins-associated with cancer cell migration by ZER and ZER-LCNs. After treatment of A549 cells with 50 µM ZER, 2.5 µM ZER-LCNs, or 5 µM ZER-LCNs for 24 h, the proteins were extracted and the expression of proteins-associated with cell proliferation was evaluated with proteome profiler human oncogenic array kit. The figure shows the protein expression of DKK1 (**A**), CAPG (**B**), CTSS (**C**), CTSB (**D**), CTSD (**E**), and PlAU (**F**). The data are expressed as mean ± SEM of 3 independent experiments. *p<0.05, **p<0.01, and ***p<0.001

Both 2.5 and 5 µM doses of ZER-LCNs significantly downregulated the protein expression of dickkopf1 (DKK1), actin capping protein, gelsolin like (CAPG), cathepsin S (CTSS), cathepsin B (CTSB), and cathepsin D (CTSD), as compared to the control (Fig. 10A–E), while only 5 µM ZER-LCNs could downregulate the protein expression of urokinase-type plasminogen activator (PLAU) (Fig. 10F, p<0.05 against the control). ZER-treated A549 cells also showed downregulation of DKK1, CAPG, CTSS, CTSB, and CTSD (Fig. 10A-E). 5 µM ZER-LCNs showed greater potential to inhibit migration than 50 µM ZER, which is highlighted by greater downregulation of DKK1 and CTSB in the presence of 5 µM ZER-LCNs relative to 50 µM ZER (Fig. 10A-D).

## Discussion

Our *in vitro* investigation in A549 cell line has demonstrated the beneficial anti-cancer activity of ZER-LCNs. We have shown that ZER-LCNs remarkably inhibits key processes of cancer progression proliferation and migration, and downregulate the various protein expression linked with cancer progression. The results of the present study indicate that ZER-LCNs provide superior anti-cancer potency and efficacy through increased inhibition A549 cell proliferation and migration at 10- to 20-fold lower concentrations of free ZER used. Mechanistically, ZER-LCNs inhibited cancer cell proliferation and migration by upregulating the mRNA levels of *P53* and *PTEN*, downregulating the mRNA levels of *KRT18*, as well as by downregulating the levels of several proteins involved in the promotion of cancer cell proliferation, migration, and invasion.

Despite a wide range of promising pharmacological benefits of ZER, it has several limitations, including low dissolution rates, poor gastrointestinal absorption and low oral bioavailability, owing to the fact that it is very slightly soluble in water, thus limiting its clinical applications (Devkota et al. 2021). To overcome these shortcomings, it is essential to develop alternative drug delivery system for ZER utilizing nanotechnology and advanced pharmaceutical approach. The encapsulation of molecules within nanoparticles is advantageous because, besides improving solubility, it improves the stability and cellular uptake of the loaded molecules, simultaneously allowing selective cell targeting (Ng et al. 2020; Paudel et al. 2022). This results in an increased therapeutic efficacy of encapsulated drugs, with substantially lower doses necessary to achieve therapeutic effect compared to free, non-encapsulated drug, and subsequently reduced adverse effects (Paudel et al. 2022). A few studies have shown that formulating ZER with cyclodextrin (Hassan et al. 2020) and nanostructured lipid carriers (Foong et al. 2018) improves its solubility and bioavailability. Pharmaceutical formulations using nanoparticles have been suggested and developed for a better and more effective drug delivery system for ZER as an anti-cancer agent. LCN drug delivery system is one of such nanotechnology-based systems that offers versatility in designing and delivery of therapeutic moiety to manage chronic respiratory diseases (Chan et al. 2021). We have previously shown that rutin-, naringenin-, and berberine-loaded LCNs offer better anti-cancer activity along with activity against oxidative stress and inflammation than using powder form of these compounds (Alnuqaydan et al. 2022; Alnuqaydan et al. 2022; Mehta et al. 2021; Mehta et al. 2021; Paudel et al. 2022; Paudel et al. 2022; Paudel et al. 2020; Paudel et al. 2021; Wadhwa et al. 2021). Continuing with the similar research hypothesis, we have formulated ZER-LCNs and investigated its anticancer potential.

In this study, the formulation of ZER using the monoolein (MO)-based LCN technology was successfully produced by dissolving the drug into the MO and then adding it to the chosen solubilizer which was P407. MO or also known as glyceryl monooleate is an amphiphilic molecule that is generally recognized as safe (GRAS) status by the FDA. It can self-assemble itself in environments that are made of aqueous surrounding because of intermolecular forces, thus allowing it to produce a liquid crystalline structure that consist of hydrophobic and hydrophilic sections inside of the lipid core (Loo et al. 2020). MO has rather low cytotoxicity when used in nanoparticles in previous studies (Bode et al. 2013; Leesajakul et al. 2004) in comparison to phytantriol-based LCNs (Hinton et al. 2014). However, MO does have the tendency to cause haemolysis when distributed through the intravenous route. Therefore, in order to reduce the likeliness of this occurring, it was proposed that the addition of P407 stabilizer would be useful as it can play the role of giving steric stabilization and coverage to the outer surfaces of the dispersed colloidal particles. This would in turn also reduce the immediate exposure of MO to the red blood cells (Loo et al. 2020).

The mean diameter of LCN is usually between 70 and 130 nm (Lee et al. 2016), whereas the ZER-LCN had a mean diameter of 180.6 ± 3 nm which also corresponds with the addition of ZER into the formulation thus slightly increasing the overall mean size. Additionally, the PDI obtained for the ZER-LCN were all negative values of less than 0.4 which indicates that the sample had low polydisperse and an overall more uniform sample (Danaei et al. 2020). Additionally, high entrapment efficiency was also observed for the ZER-LCN formulation at 90.63 ± 0.13%. The entrapment efficiency is the difference between the initial drug and the free drug in the supernatant with respect to the total amount added into the nanoformulation. The in vitro drug release study is an important study for the identification of the efficacy, safety as well as the quality of nanoparticle-based drug delivery systems (Weng et al. 2020). Our results suggested that the ZER-LCN was able to release a larger amount of drug in comparison to pure ZER. Additionally, a sustained release of the drug for over 24 h was observed and this prolonged release is an added benefit as it ensures that patients would reap the benefits of the safe drug dosage over a longer period as well as being able to reduce the frequency of the drug consumption.

Once we confirmed that our ZER-LCNs exert favorable characterization data in terms of particle size, polydispersity index, entrapment efficiency, and in vitro release, we proceeded with the investigation of in vitro biological activity against A549 cells. The two key events of tumorigenesis are (a) proliferation and (b) migration/metastasis, which are governed by several pathways, and transcription and expression of cancer-related genes and proteins, respectively. P53 is a nuclear protein whose main physiological role is to regulate the progression of cells through the cell cycle, arresting the cell cycle in case of DNA damage, and initiating apoptosis if the damage is irreparable (Chasov et al. 2021). Loss-of-function mutations of p53 are very frequent in different types of cancer (Jin et al. 2010). This makes p53 an intriguing option as a therapeutic target, with many approaches emerging recently in an attempt to restore its function (Hassin and Oren 2022). Another important tumor suppressor whose function is often loss in many cancers, including LC, is PTEN (Gkountakos et al. 2019; Jin et al. 2010). The PTEN protein is a potent oncosuppressor whose physiological function is to maintain the homeostasis of the mitogenic Phosphatidylinositol-3-kinase (PI3K)/AKT pathway by dephosphorylating phosphatidylinositol-3, 4, 5-phosphate (PIP3) (Jin et al. 2010; Molinari and Frattini 2013). The loss of expression and/or function of oncosuppressors such as p53 and PTEN results, through different pathways, in increased cell proliferation and reduced cell death (Jin et al. 2010). This is often accompanied by the aberrant expression of specific proteins that may be used as a cancer biomarker such as KRT18 (Zhang et al. 2019).

Our cell proliferation assay revealed that ZER-LCNs remarkably inhibited the A549 proliferation by ~45% at a dose of 5 µM (Fig. 4). Interestingly, the pure ZER upto the dose of 50 µM did not significantly change the cell proliferation indicating ZER-LCN is more potent than its pure form. Furthermore, this was supported by our colony formation assay where ZER-LCNs at a dose of 5 µM inhibited the colony formation of A549 compared to both control (only media treated) and pure ZER at 50 µM (Fig. 5). Mechanistically, we have revealed that the decrease in A549 cell proliferation was due to the inhibition of protein expression of Axl, progranulin, Survivin/BIRC5, and ErbB1/HER1 that are involved in cell proliferation/growth (Fig. 9). The expression of oncogene Axl is observed in 60% of NSCLC cell line and it is known to facilitate tumor cell growth and adhesion (Kim et al. 2015; Wimmel et al. 2001). *Progranulin* expression is correlated with A549 growth and induce tumor growth in vivo (He and Bateman 1999). Survivin/BIRC5 inhibits apoptosis of tumor cells, and therefore supports cancer cell survival (Hirano et al. 2015; Schott et al. 1995). HER1 is a member of epidermal growth factor receptor (EGFR) family with crucial function of mediating NSCLC proliferation (Guo et al. 2016). *P53* and *PTEN* are well known tumor suppressor genes in NSCLC (Andjelkovic et al. 2011). It is interesting to note that tumor suppressor gene *P53* and *PTEN* were upregulated by only ZER-LCN but not by pure ZER (Fig. 8).

**Fig. 11:**
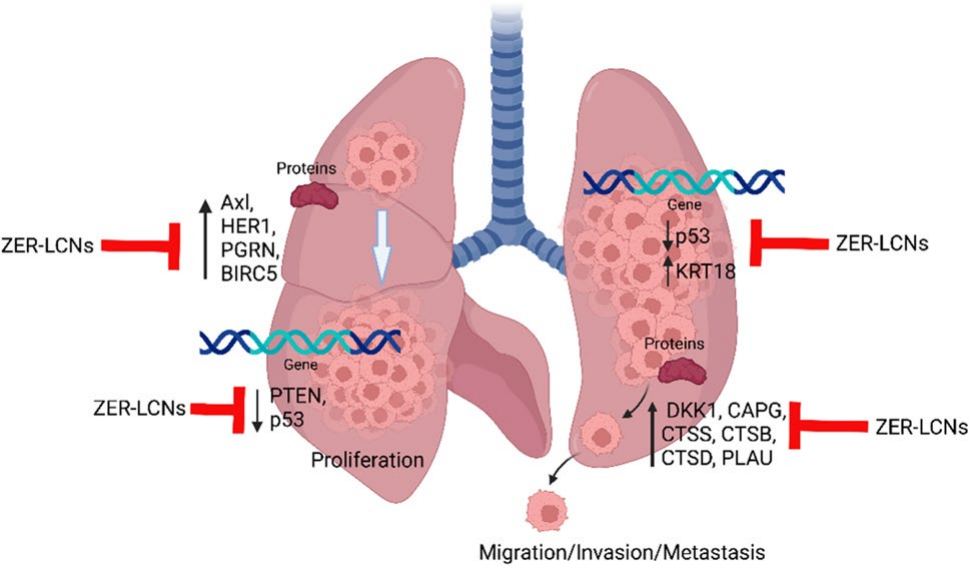
Anticancer mechanism of action of ZER-LCNs in vitro in A549 cells. (Abbreviations: ZER-LCNs, Zerumbone-liquid crystalline nanoparticles; p53, tumor protein 53; KRT18, keratin 18; PTEN, phosphatase and tensin homolog; Axl, AXL receptor tyrosine kinase; HER1, epidermal growth factor receptor; PGRN, progranulin; BIRC5, Baculoviral IAP repeat containing 5; DKK1, Dickkopf1; CAPG, actin capping protein, gelsolin like; CTSS, Cathepsin S; CTSB, Cathepsin B; CTSD, Cathepsin D; PLAU, urokinase-type plasminogen activator)

In relation to A549 migration, we have observed that ZER-LCNs drastically inhibits the A549 would closure in scratch wound healing assay and trans-well chamber assay, the methods to quantify cell migration. This was evident by ~27% inhibition of wound closure by 5 µM ZER-LCNs while pure ZER could not significantly close the wound compared to the untreated control (Fig. 6). Similarly, in trans-well chamber assay, 5 µM ZER-LCNs inhibited the migration of A549 by ~60% while pure ZER could only inhibit ~32% (Fig. 7). Mechanistically, we have revealed that the decrease of A549 cell proliferation was due to the inhibition of protein expression of CTSB, CTSD, CTSS, DKK1, CAPG, and PLAU that are involved in cell migration/metastasis (Fig. 10). CTSS protein is associated with NSCLC progression as CTSS can cleave proteoglycan of interstitial matrix such as decorin (Kehlet et al. 2017) and nidogen-1 (Willumsen et al. 2017) to facilitate the invasion of NSCLC cell. DKK1, CTSB, CTSD, and CAPG are also associated with the metastatic potential of cancer (Shao et al. 2011; Tan et al. 2013; Zhang et al. 2017). Our biological study clearly suggests that ZER-LCNs possess more significant anti-cancer potential against A549 cells compared to pure ZER. *KRT18* gene is overexpressed in human cancer and positively correlated with tumor invasion/metastasis (Zhang et al. 2014). In our study, we observed that *KRT18* gene expression was significantly downregulated by ZER-LCNs but not by pure ZER. These findings are diagrammatically summarized in Fig. 11.

We cannot neglect the various limitations in our study that could be a research platform for us and other researcher for further expansion of our current study. Our study lacks the inclusion of the expression of other lung cancer related genes besides *P53*, *PTEN*, and *KRT18*. It would be interesting to explore further to validate whether the genes encoding the proteins in Figs. 9 and 10 are inhibited by ZER-LCNs. As our research outcomes is based on in vitro experimental data, there is a scope for further expansion of our hypothesis utilizing in vivo animal models of lung cancer. Furthermore, apart for the A549 cell line, there are many other cells line of both NSCLC and SCLC where we can study the therapeutic potential of ZER-LCNs as anticancer agents. Moving forward, it would be useful and compelling to conduct in vivo biological analysis of ZER in the LCN formulation through the inhalation delivery method in experimental mice model with other models that have been established. Using an animal model would also allow us to determine the long-term safety, pharmacokinetics, herb-drug complexities, and many other factors as these plays a pivotal part in determining toxicity and the bioavailability of drugs. Nevertheless, our study suggests that with further strengthening of research with Zerumbone -LCNs, it can be a promising therapeutic option for lung cancer.

## Conclusion

In conclusion, ZER-LCNs show significant anti-proliferative and anti-migratory activity in A549 cells by regulating the mRNA expression of *P53*, *PTEN*, and *KRT18* and the expression of several proteins associated with cell proliferation and migration such as AXL, HER1, PGRN, BIRC5, DKK1, CAPG, CTSS, CTSB, CTSD, and PlAU. This study highlights successful application of nanotechnology in formulating LCN-encapuslated ZER to generate superior efficacy and potency compared to free ZER for the treatment of NSCLC. Formulating ZER into LCNs poses much greater pharmacological and biological benefits in comparison to free ZER on its own. The potent biological activities of ZER-LCN prove that it is able to overcome many of the unfavorable characteristics of pure ZER using the nanoformulation method. This study will help to direct future research towards the investigation and development of ZER as a potential anti-cancer drug therapy with the help of nanotechnology.

## Data Availability

The data and materials that support the findings of this study will be made available by the corresponding author, upon reasonable request.

## Abbreviations

ZER: Zerumbone
LCN: Liquid crystalline nanoparticle
